# A mixed methods evaluation of the current state of perinatal mental healthcare and users’ acceptability of a digital assessment for perinatal mental health

**DOI:** 10.1192/j.eurpsy.2021.1845

**Published:** 2021-08-13

**Authors:** B. Spadaro, N. Martin-Key

**Affiliations:** Cambridge Centre For Neuropsychiatric Research, Department Of Chemical Engineering And Biotechnology, University of Cambridge, Cambridge, United Kingdom

**Keywords:** Digital Mental Health, Maternal mental health, Perinatal Mental Health, Paternal mental health

## Abstract

**Introduction:**

Perinatal mental health symptoms commonly remain underdiagnosed in maternity care settings in the UK, with the COVID-19 pandemic having further disrupted access to adequate care provision. Digital technologies may offer innovative ways to support the mental health needs of women and partners and assist midwives in recognition of concerns.

**Objectives:**

We set to investigate the current state of perinatal mental healthcare provision in the UK and the acceptability of a digital mental health assessment.

**Methods:**

The study entailed completing an online survey. 829 women, 103 partners, and 90 midwives participated in the study. Quantitative data were explored using descriptive statistics. Open-ended responses regarding the perceived benefits and barriers to using a digital mental health assessment were investigated using thematic analysis. Resultant themes were then mapped onto the Capability, Opportunity, and Motivation Model of Behaviour (COM-B model).

**Results:**

The provision of perinatal mental healthcare support was limited and varied across respondents, particularly throughout the COVID-19 pandemic. There was a strong interest in using a digital mental health assessment placed within maternity healthcare settings to screen, diagnose, and triage concerns (Figure 1). In-person and blended care approaches (i.e., in-person and remote support) were preferred by women and partners in the event of further care being advised (Figure 1). Identified barriers and benefits mainly related to physical opportunity (e.g., accessibility), psychological capability (e.g., cognitive skills) and automatic motivation (e.g., emotions).

**Figure:**
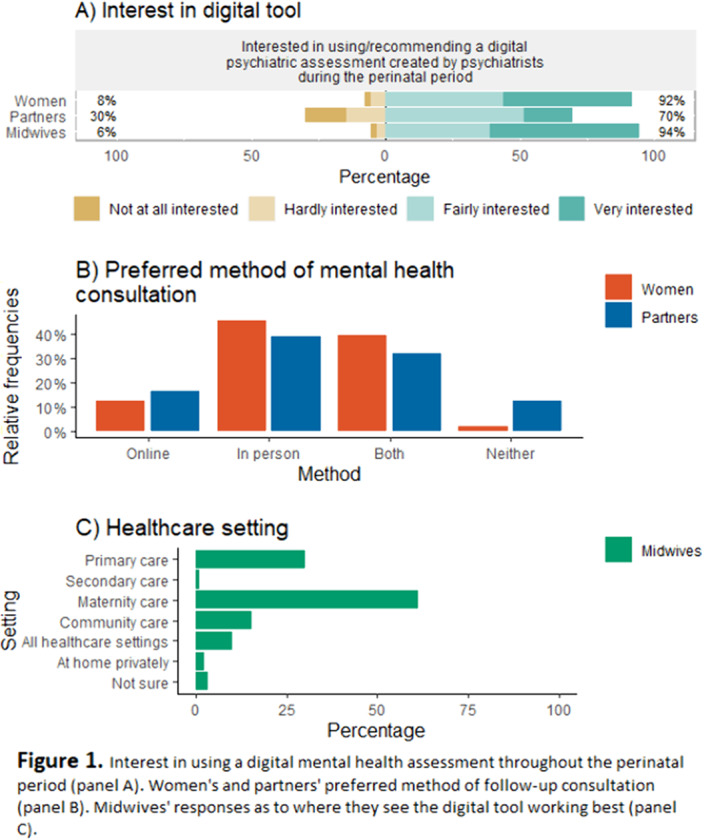

**Conclusions:**

This study provides proof-of-concept support for the development of a digital mental health assessment to inform clinical decision-making for perinatal mental health concerns.

**Disclosure:**

NMK has financial interest in Psyomics Ltd., a company developing digital diagnostic devices for neuropsychiatric disorders.

